# Sweet Syndrome With Painless Skin Lesions and Myopericarditis: A Case Report and Literature Review

**DOI:** 10.1002/ccr3.70153

**Published:** 2025-02-26

**Authors:** Fakhruddin Almuzghi, Islam Elzouki, Moaz O. Moursi, Asiya Aqeel Thakur, Mahir Petkar

**Affiliations:** ^1^ Department of Internal Medicine Hamad Medical Corporation Doha Qatar; ^2^ Department of Laboratory Medicine and Pathology Hamad Medical Corporation Doha Qatar

**Keywords:** acute febrile neutrophilic dermatosis, case report, Myopericarditis, skin lesion, sweet syndrome

## Abstract

Sweet syndrome is a rare inflammatory disease that typically presents with painful erythematous skin lesions and fever. Although the skin lesions are generally painful on clinical examination, in this case, they were noted to be painless without tenderness. Moreover, our case is unique in that this patient had myopericarditis, a rare extracutaneous manifestation of sweet syndrome.

## Graphical abstract

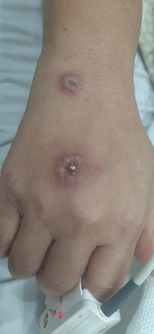

## 1 Introduction

Sweet syndrome, also known as acute febrile neutrophilic dermatosis, is a rare inflammatory disorder that presents with fever, leukocytosis, and painful skin lesions. Skin lesions can be erythematous papules, nodules, or plaques. This rare disorder can have many extracutaneous manifestations involving organs like the heart, eyes, brain, kidney, muscles, and bones and was first described by Robert Douglas Sweet in 1964 [1]. This uncommon disorder is typically associated with infections like upper respiratory tract infections, autoimmune diseases like inflammatory bowel disease, or pregnancy [2, 3]. Sweet syndrome can be associated with malignancies like leukemia or breast cancer [4, 5]. It can also be associated with the use of some medications, such as trimethoprim‐sulfamethoxazole [2]. Therefore, Sweet syndrome can be classified into three categories on the basis of etiology: Classical Sweet syndrome, malignancy‐associated Sweet syndrome, and drug‐induced Sweet syndrome. Patients with Sweet syndrome might look severely ill. The onset of painful skin lesions may be immediate or days to weeks before or after the onset of fever. This case report presents an atypical feature of the classical skin lesion seen in patients with Sweet syndrome. Our case is unique in that our patient did not complain of pain or tenderness upon the palpation of the skin lesions, which are typically known to be painful.

## 2 Case History/Examination

A 47‐year‐old Filipino lady presented with a new onset of fever for 4 days. She sought medical attention at a primary health care center. She was prescribed amoxicillin‐clavulanic acid, vitamin C, and paracetamol. However, she continued to spike high‐grade fever and developed a new onset headache, sweating, chills, vomiting, malaise, and abdominal pain. Her vomitus was non‐bilious and occurred ten times. The headache, vomiting, and abdominal pain began after she took antibiotics and resolved after she discontinued them. She had a poor appetite but no hematemesis, melena, or change in bowel habits. She had no cough, shortness of breath, upper respiratory tract symptoms, hemoptysis, chest pain, palpitations, or arthralgia. There was no history of loss of consciousness, confusion, ear pain, tooth pain, or visual or auditory abnormalities. She had a regular menstrual cycle and denied any genitourinary symptoms. She noticed two painless skin lesions on the dorsal aspect of her right hand. The first lesion appeared 2 weeks before the fever onset, and the second lesion appeared a few days after. She denied insect or animal bites and had no history of trauma or skin rash. In terms of past medical history, she was diagnosed with gastroesophageal reflux disease in her home country and was managed by antacids. Family history revealed a stroke diagnosed in her father; however, she had no family history of cancer. She is married and is working as a housemaid. She traveled from the Philippines to Qatar 2 months before the hospital presentation.

**FIGURE 1:**
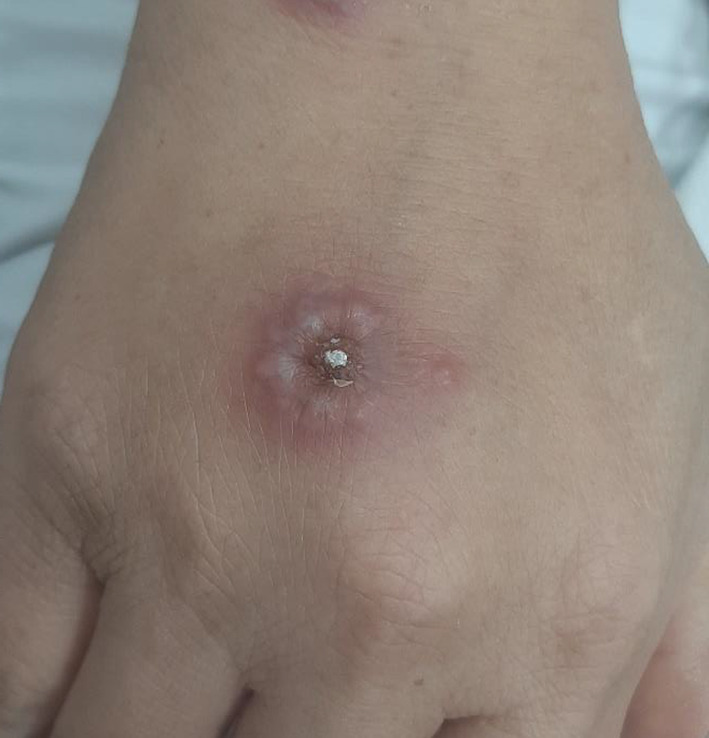
Two erythematous typical Sweet syndrome skin lesions on the dorsal surface of the right hand.

Upon physical examination, she was febrile, with a temperature of 39.1°C, and tachycardic, with a regular pulse rate of 144 beats per minute. Her blood pressure was 111/78 mmHg, and her respiratory rate was 25 breaths per minute. Two painless erythematous skin lesions were present on the dorsal surface of the right hand (Figure 1). No palpable lymph nodes, mouth ulcers, dental caries, or conjunctivitis was noted upon physical examination. Chest auscultation revealed bilateral air entry without crepitation or wheezing. Heart sounds were distant, with normal S1 and S2. The abdomen was soft, lax, and non‐tender, and normal bowel sounds were present. No jaundice, arthritis, rash, lower limb edema, or neck stiffness was noted. There were no signs of meningeal irritation or neurological deficit, and she was alert and oriented.

## 3 Methods (Investigations)

Laboratory blood investigations revealed elevated white blood cell counts, mainly neutrophils, and an elevated C‐reactive protein. Peripheral blood smear showed neutrophilic leukocytosis with mild toxic features and atypical lymphocytes. Alkaline phosphatase, gamma‐glutamyl transferase, alanine transaminase, and aspartate transferase were slightly elevated. However, bilirubin, lipase, and amylase were within the normal limits. Troponin was significantly raised. Screening for antinuclear antibodies was negative (Table 1).

**TABLE 1:** Initial blood workup.

Hematology	
White blood cell count	17.7 × 10^3^/μL (Normal range: 4–10)
Red blood cell count	3.8 × 10^3^/μL (Normal range: 3.8–4.8)
Hemoglobin	11.2 g/dL (Normal range: 12–15)
Hematocrit	34.2% (Normal range: 36–46)
Mean corpuscular volume	80.5 fL (Normal range: 83101)
Platelets	304 × 10^3^/μL (Normal range: 150–410)
Absolute neutrophil count #	14 × 10^3^/μL (Normal range: 2–7)
Lymphocytes #	1.4 × 10^3^/μL (Normal range: 1–3)
Monocytes #	0.2 × 10^3^/μL (Normal range: 0.2–1)
Eosinophils #	0.31 × 10^3^/μL (Normal range: 0.02–0.5)
Neutrophils %	88%
Lymphocytes %	9%
Monocytes %	1%
Eosinophils %	2%
Morphology Comment	Neutrophilic leukocytosis with toxic features. Few reactive lymphocytes.
Peripheral Smear	Red cells: Mild anemia, few ovalocytes, spherocytes, mild polychromatophilic cells, mild rouleaux formation. Leukocytes: Mild neutrophilic leukocytosis with mild toxic features. Few reactive and occasional atypical lymphocytes. Platelets: Adequate, normal
**Blood chemistry**	
Urea	3.5 mmol/L (Normal range: 2.5–7.8 mmol/L)
Creatinine	44 μmol/L (Normal range: 62–106 μmol/L)
Sodium	136 mmol/L (Normal range: 13.3–146 mmol/L)
Potassium	3.2 mmol/L (Normal range: 3.5–5.3 mmol/L)
Chloride	100 mmol/L (Normal range: 95–108 mmol/L)
Bicarbonate	25 mmol/L (Normal range: 22–29 mmol/L)
Total bilirubin	12 μmol/L (Normal range: 0–21)
Total protein	67 g/L (Normal range: 60–80)
Albumin	25 g/L (Normal range: 35–50)
Alkaline phosphatase	325 U/L (Normal range: 35–104 μ/L)
Alanine transaminase	64 U/L (Normal range: 0–40)
Aspartate aminotransferase	31 U/L (Normal range: 0–40)
Troponin I	975 ng/L (Normal range: 3–10 ng/L)
C‐Reactive Protein (CRP)	333.8 mg/L (Normal range: 0–5)
NT pro‐BNP	4578 pg/mL (Normal range: < 300 pg/mL)

Testing for tuberculosis, hepatitis B, Hepatitis C, syphilis, Cytomegalovirus, Ebstein‐Barr Virus, Herpes, Measles, Parvovirus B19, Rubella, Varicella zoster, and Human Immunodeficiency Virus was negative. The viral panel was also negative (Table 2). Sputum and blood cultures showed no growth of organisms, and urinalysis showed no evidence of a urinary tract infection.

**TABLE 2:** Viral panel.

Respiratory (Nasopharyngeal)	
COVID‐19 PCR	Negative
Influenza Virus A PCR	Negative
Influenza Virus B PCR	Negative
Respiratory syncytial virus PCR	Negative
**Blood**	
Adenovirus PCR	Negative
CMV antibody IgG	Reactive
CMV antibody IgM	Non‐reactive
EBV Capsid antigen IgG	Positive
EBV Capsid antigen IgM	Equivocal
Enterovirus PCR	Negative
Hepatitis B surface antigen	Non‐reactive
Hepatitis B surface antibody	Non‐reactive
Hepatitis B core antibody	Non‐reactive
Hepatitis C antibody	Non‐reactive
Herpes Simplex Virus (HSV1) PCR	Negative
Herpes Simplex Virus (HSV2) PCR	Negative
Herpes Simplex Type I IgG	Positive
Herpes Simplex Type II IgG	Negative
HIV Ag/Ab Combo	Non‐reactive
Measles IgG antibody	Positive
Measles IgM antibody	Negative
Mumps Ab IgG	Positive
Mumps Ab IgM	Negative
Mumps Virus PCR	Negative
Orthopox Virus PCR	Negative
Parecho Virus PCR	Negative
Parvovirus B19 antibody IgG	Positive
Parvovirus B19 antibody IgM	Negative
Rubella antibody IgG	Reactive
Rubella antibody IgM	Non‐reactive
Varicella Zoster antibody IgG	Positive
Varicella Zoster antibody IgM	Negative
Varicella Zoster Virus PCR	Negative

**FIGURE 2:**
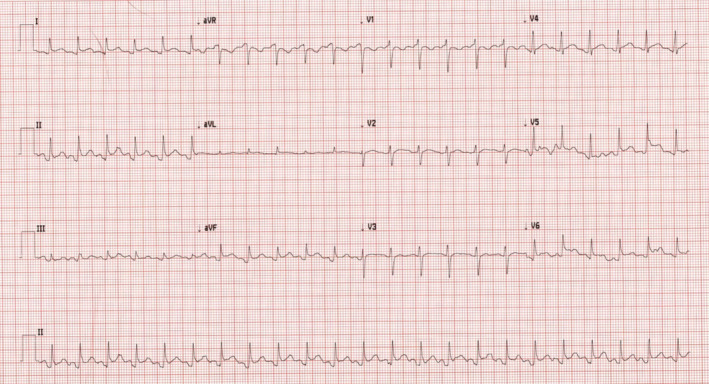
12‐lead ECG showing PR‐segment depression and ST‐segment elevation in most of the leads, indicative of pericarditis.

**FIGURE 3:**
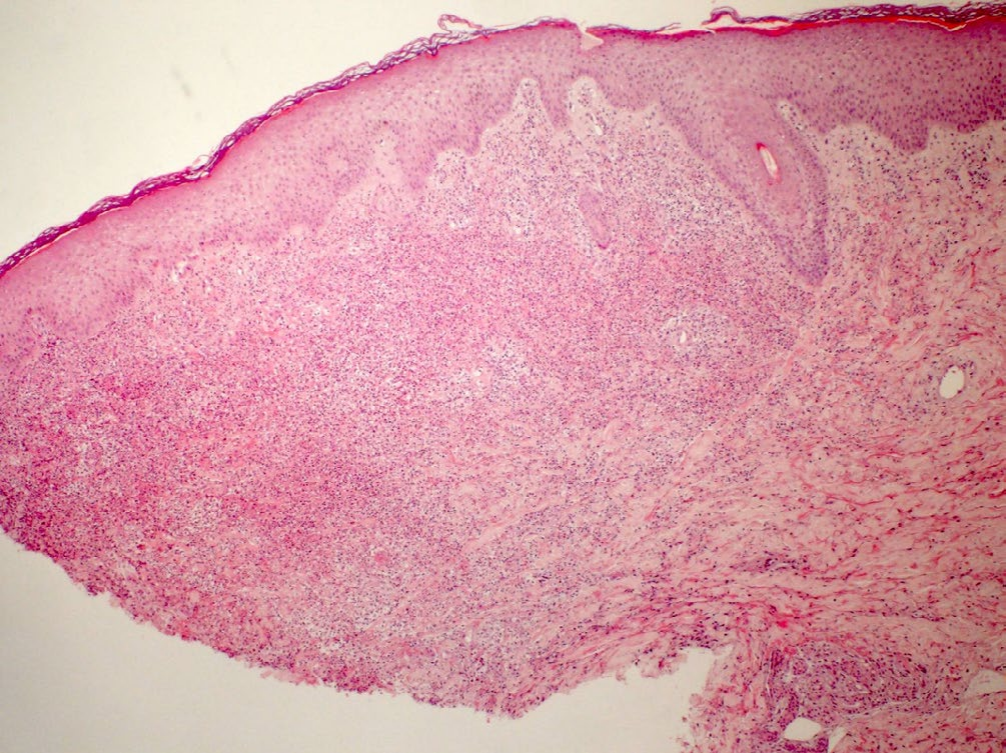
Skin punch biopsy showing normal‐appearing orthokeratotic epidermis with underlying papillary edema and intense diffuse dermal neutrophilic inflammatory infiltrate, expanding the reticular dermis (H and E × 40).

**FIGURE 4:**
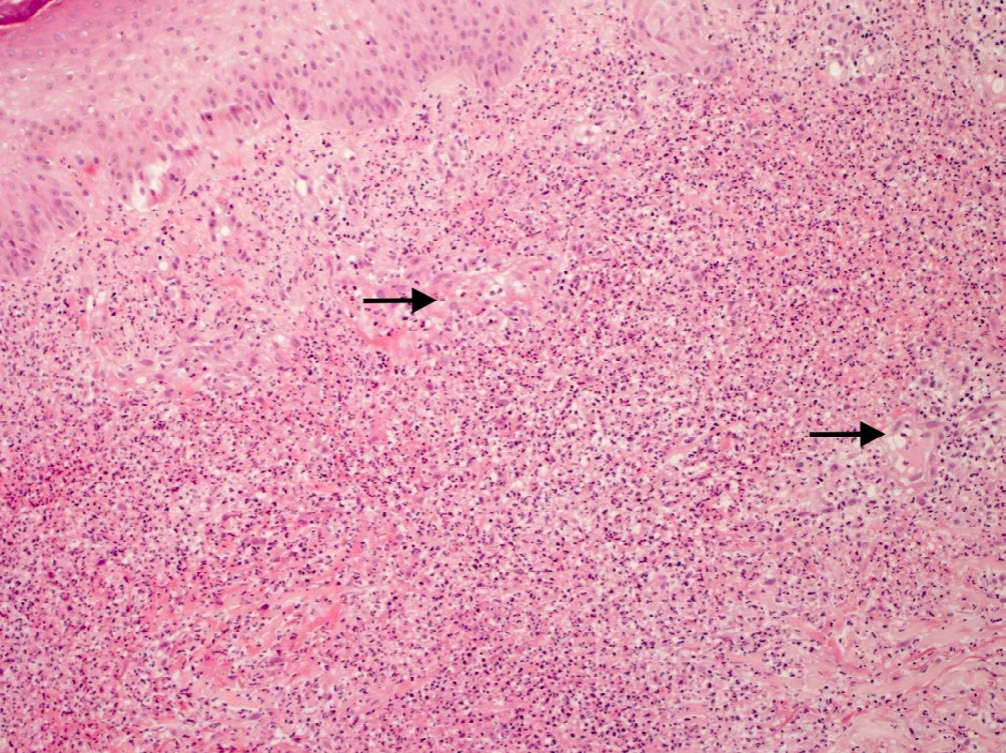
Higher power view of the dermal infiltrate. Blood vessels amidst the inflammation display endothelial swelling (black arrows), but there are no features of vasculitis (H and E × 100).

**FIGURE 5:**
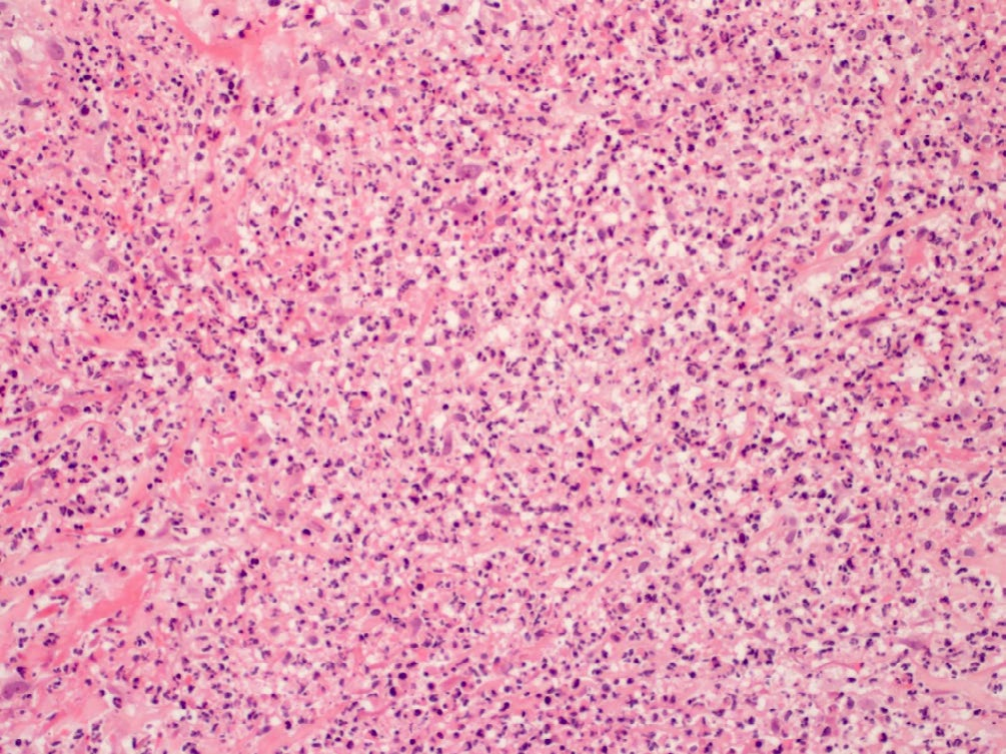
Leukocytoclastic nuclear debris is noted within the dense inflammatory infiltrate (H and E × 200).

Chest X‐ray was unremarkable. Electrocardiogram demonstrated sinus tachycardia with a heart rate of 137 beats per minute along with a PR‐segment depression and ST‐segment elevation in most leads (Figure 2). Transthoracic echocardiography showed normal left ventricle size but mildly reduced left ventricle systolic function was noted with an ejection fraction of 42%. Mild global hypokinesis of the left ventricle was present. Right ventricle function was also moderately reduced, but right ventricle size was normal. The left and right atriums were normal in size, and no valvular abnormalities, regional wall motion abnormalities, or pericardial effusion was detected. CT pulmonary angiogram scan showed no evidence of pulmonary embolism. An abdominal ultrasound scan was unremarkable. Skin punch biopsy of the right‐hand lesion showed a relatively unremarkable epidermis. There was prominent papillary dermal edema with extensive diffuse dermal neutrophilic inflammation (Figures 3, 4, and 5). Leukocytoclastic nuclear debris was present interstitially. Endothelial swelling was noted, but features of vasculitis, such as fibrin deposition in the vessel walls, were not observed. The histological appearances were consistent with acute febrile neutrophilic dermatosis (Sweet syndrome).

## 4 Conclusion and Results (Outcome and Follow‐Up)

The patient was diagnosed with Sweet syndrome with myopericarditis. By the time the biopsy result was out, the patient had already been treated with a course of antibiotics for a suspected lower respiratory tract infection and corticosteroids for myopericarditis. She showed a significant clinical improvement in her presenting symptoms. The patient did not attend her scheduled follow‐up appointments after discharge, limiting further evaluation of her long‐term outcomes.

## 5 Discussion

Sweet syndrome is a rare inflammatory dermatological disease distinguished by fever and skin lesions. Skin lesions are caused by the infiltration of neutrophils in the dermis. Its clinical characteristics and presentations can vary between patients, thus making it a challenging condition to diagnose. The diagnostic criteria of Sweet syndrome include two major features and four minor features. The major features are erythematous plaques or nodules and histopathologic evidence of a dense neutrophilic infiltrate without evidence of leukocytoclastic vasculitis. The minor features are temperature > 38°C, response to systemic steroids, association with medical conditions (such as hematologic or visceral malignancy, inflammatory disease, recent upper respiratory or gastrointestinal infection, or vaccination), and blood test abnormalities at presentation (such as elevated C‐reactive protein, white blood cell counts, and erythrocyte sedimentation rate) [5].

The diagnosis of classical Sweet syndrome requires both major and two of four minor criteria [6]. Our patient fit the diagnostic criteria as she had erythematous skin lesions and neutrophilic infiltration of the dermis in addition to high‐grade fever and elevated inflammatory markers. The significant finding was the absence of tenderness of the skin lesion, and the patient's description of the skin lesion as painless. This patient denied drug intake, and the malignancy workup was negative. Therefore, it is considered a classical sweet syndrome.

Sweet syndrome can have different extracutaneous manifestations as it can affect different organ systems such as the brain (encephalitis), cardiovascular system (myocarditis, aortitis, or aortic stenosis), lung (obstruction airway disease, neutrophilic alveolitis, or pleural effusions), gastrointestinal system (hepatitis, hepatomegaly, or splenomegaly), and musculoskeletal system (sterile osteomyelitis) [7]. Our patient developed myopericarditis and mild hepatitis, which are considered a rare extracutaneous manifestation of this uncommon disorder.

This case report demonstrates an atypical clinical presentation of Sweet syndrome, characterized by painless skin lesions and the rare extracutaneous manifestation of myopericarditis. Diagnosing this condition was challenging because of the absence of tenderness in the classical skin lesions, which are typically painful, and the overlap of systemic symptoms with other more common conditions. The patient's presentation highlights the importance of applying diagnostic criteria systematically, utilizing histopathological examination, and carefully excluding mimicking disorders such as infections and autoimmune diseases. This case also highlights the potential for Sweet syndrome to present with significant systemic involvement, necessitating a multidisciplinary approach to management.

Beyond its clinical implications, this case offers significant educational value. It emphasizes the need for clinicians to remain vigilant, particularly in patients with unusual or atypical features of a well‐recognized condition. The detailed exploration of the diagnostic process, histopathological findings, and systemic implications provides a useful guide for clinicians in recognizing and managing rare manifestations of Sweet Syndrome. Additionally, it serves as a reminder of the need for thorough follow‐up in cases of rare diseases, as long‐term outcomes may offer further insights into disease progression and management.

In conclusion, this report provides a practical framework for understanding and managing Sweet syndrome in patients presenting with uncommon clinical features. It serves as a valuable educational resource for healthcare providers, enhancing awareness of the diverse presentations, diagnostic challenges, and systemic implications of this rare inflammatory disorder.

## Author Contributions

**Fakhruddin Almuzghi:** conceptualization, data curation, methodology, writing – original draft, writing – review and editing. **Islam Elzouki:** investigation, writing – original draft. **Moaz O. Moursi:** methodology, writing – review and editing. **Asiya Aqeel Thakur:** data curation, writing – original draft, writing – review and editing. **Mahir Petkar:** investigation, writing – original draft.

## Ethics Statement

All methods were performed in accordance with the relevant guidelines and regulations. This study was approved by Hamad Medical Corporation Medical Research Center (MRC‐04‐24‐458).

## Consent

Written informed consent was obtained from the patient for the publication of this case report and any accompanying images. A copy of the written consent is available for review by the editor‐in‐chief of this journal on request.

## Conflicts of Interest

The authors declare no conflicts of interest.

## Data Availability

The authors have nothing to report.
